# Supplementary material for: Controlling the motion of multiple objects on a Chladni plate
Source: Nat Commun. 2016 Sep 9;7:12764. doi: 10.1038/ncomms12764 (PMC5023966; doi:10.1038/ncomms12764)
Supplement: Supplementary Information — Supplementary Figures 1-12 and Supplementary Note 1. [file ncomms12764-s1.pdf]

Supplementary Figures

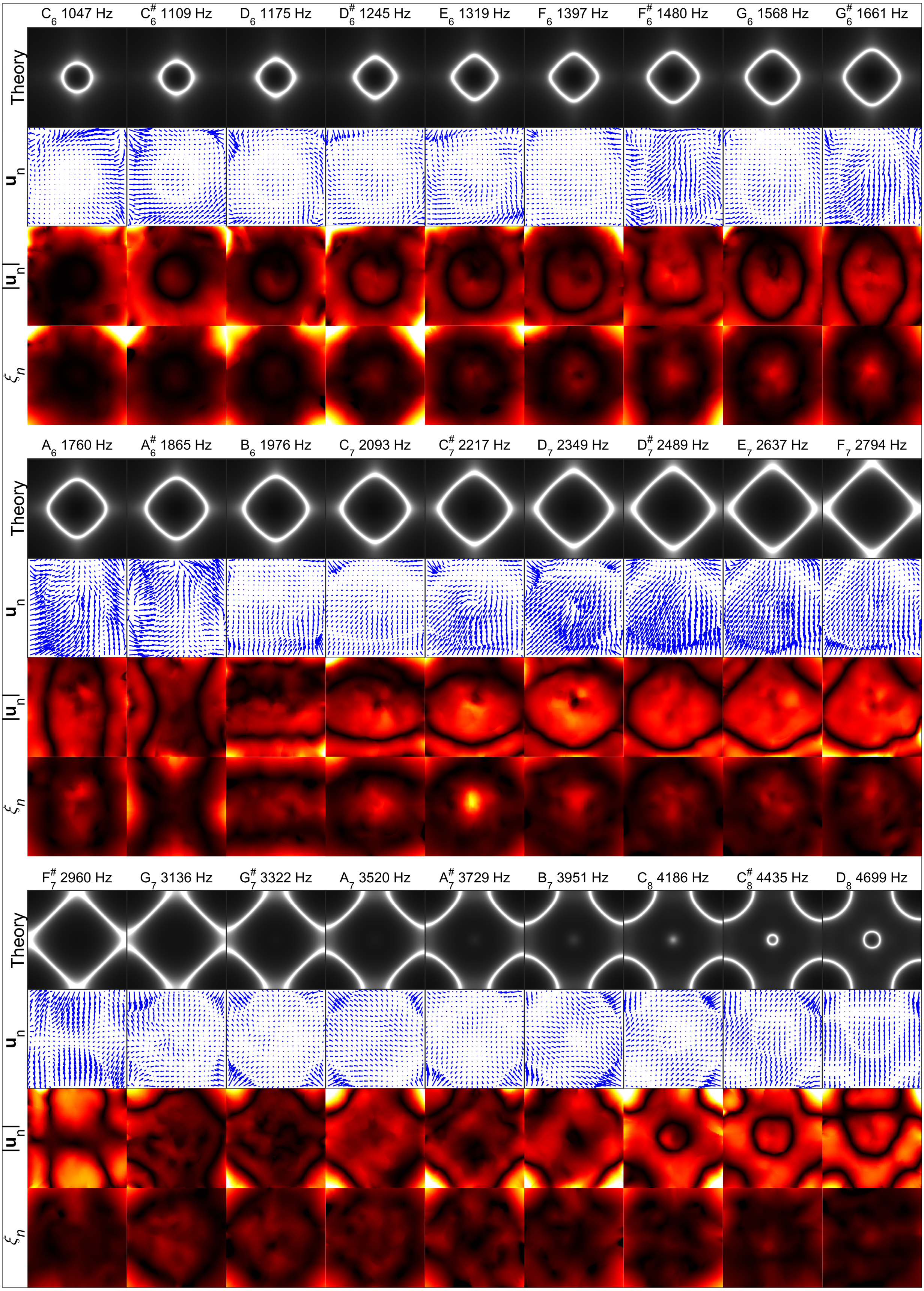

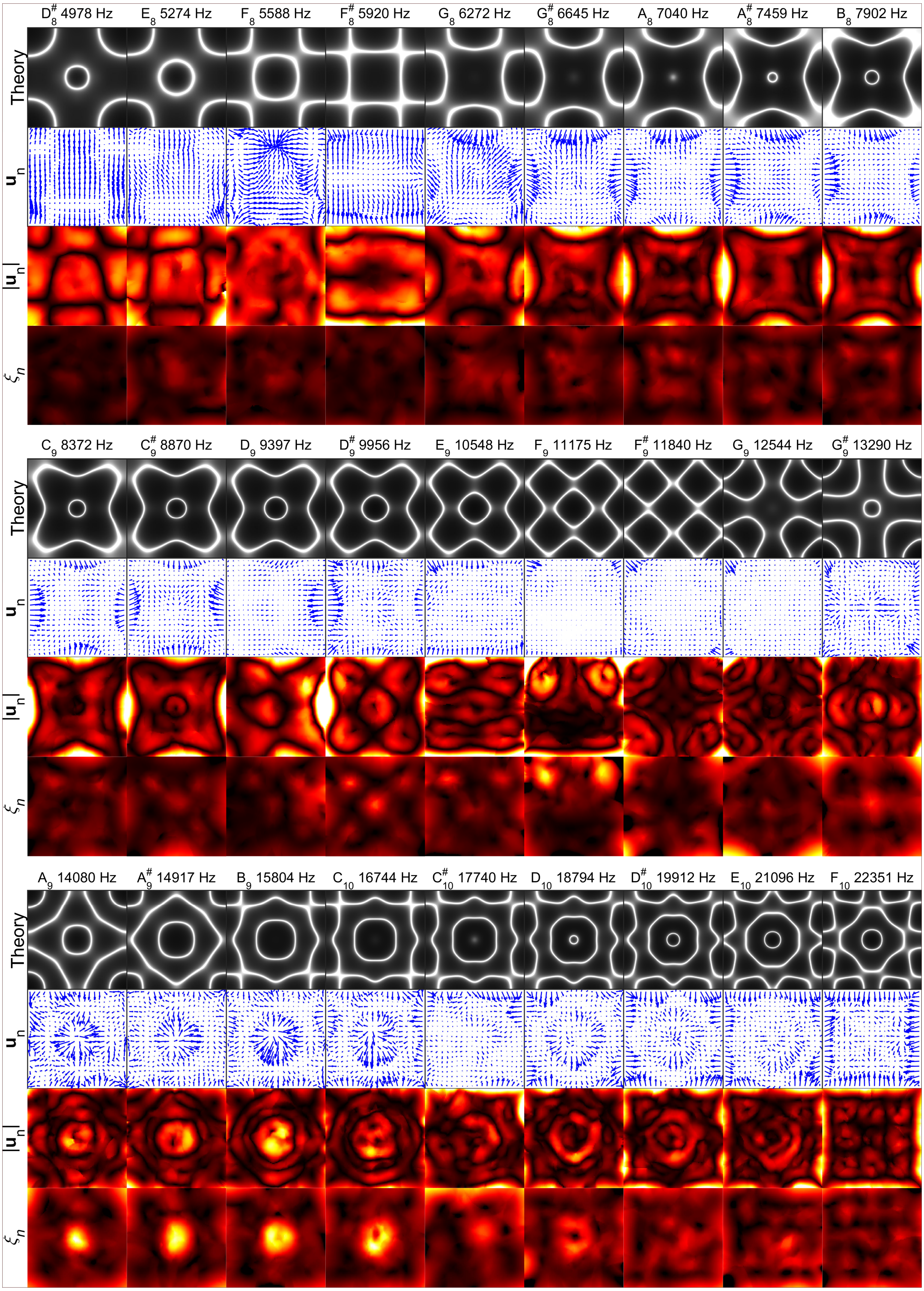
D#8 4978 Hz
E8 5274 Hz
F8 5588 Hz
F#8 5920 Hz
G8 6272 Hz
G#8 6645 Hz
A8 7040 Hz
A#8 7459 Hz
B8 7902 Hz
C9 8372 Hz
C#9 8870 Hz
D9 9397 Hz
D#9 9956 Hz
E9 10548 Hz
F9 11175 Hz
F#9 11840 Hz
G9 12544 Hz
G#9 13290 Hz
A9 14080 Hz
A#9 14917 Hz
B9 15804 Hz
C10 16744 Hz
C#10 17740 Hz
D10 18794 Hz
D#10 19912 Hz
E10 21096 Hz
F10 22351 Hz
Theory
un
|un|
ξn

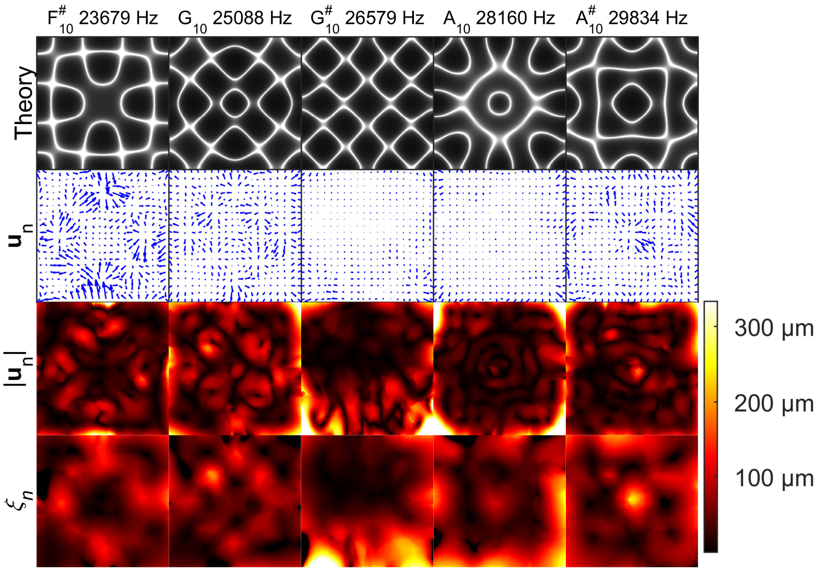

Supplementary Figure 1. Comparison of theoretical Chladni figures and statistically modeled displacement fields for the notes used in the manipulation. First row shows the theoretically computed Chladni figure, second row shows the modelled vector field $\mathbf{u}_n$, third row shows the absolute displacement $|\mathbf{u}_n|$ (units: µm) and the fourth row shows the residual ξ_n (units: µm).

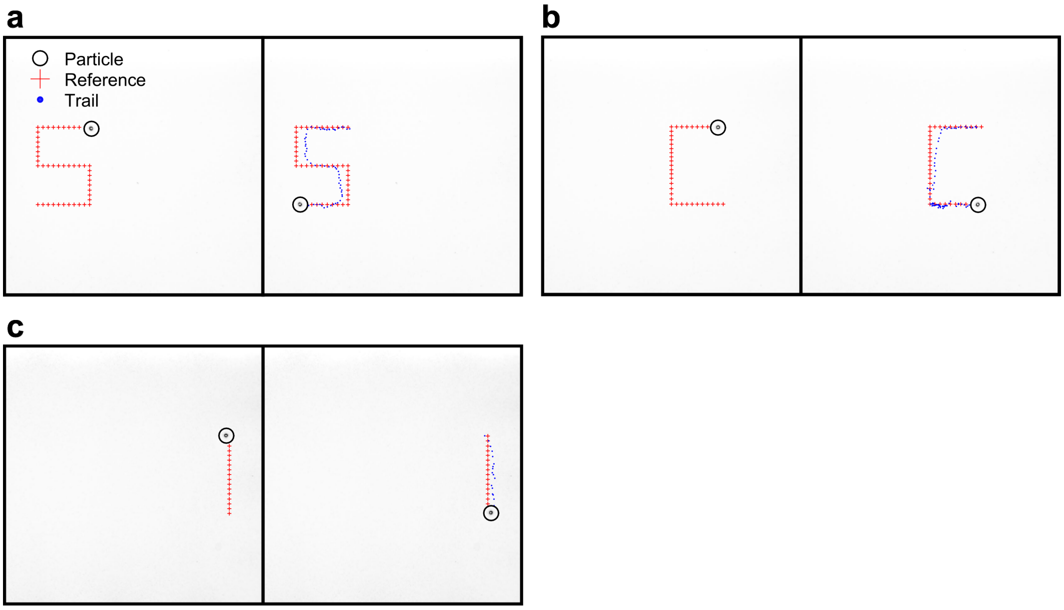

Supplementary Figure 2. Manipulation of a single particle. The same letters as in Fig. 1c were drawn in three different experiments. (**a**) Letter S. (**b**) Letter C. (**c**) Letter I.

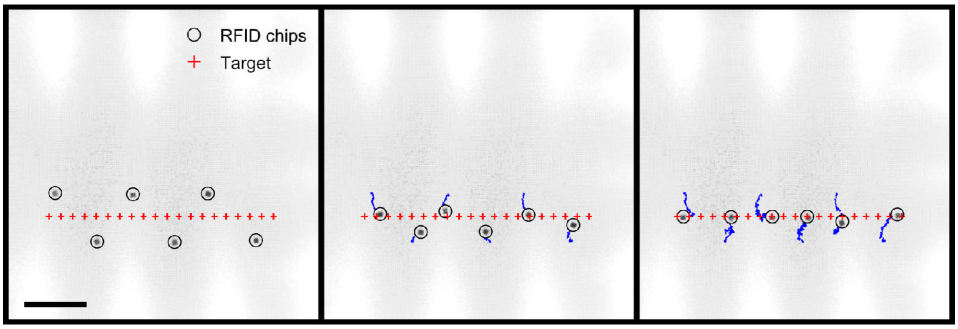

Supplementary Figure 3. Alignment of six radio frequency identification (RFID) chips. Scale bar: 1 cm.

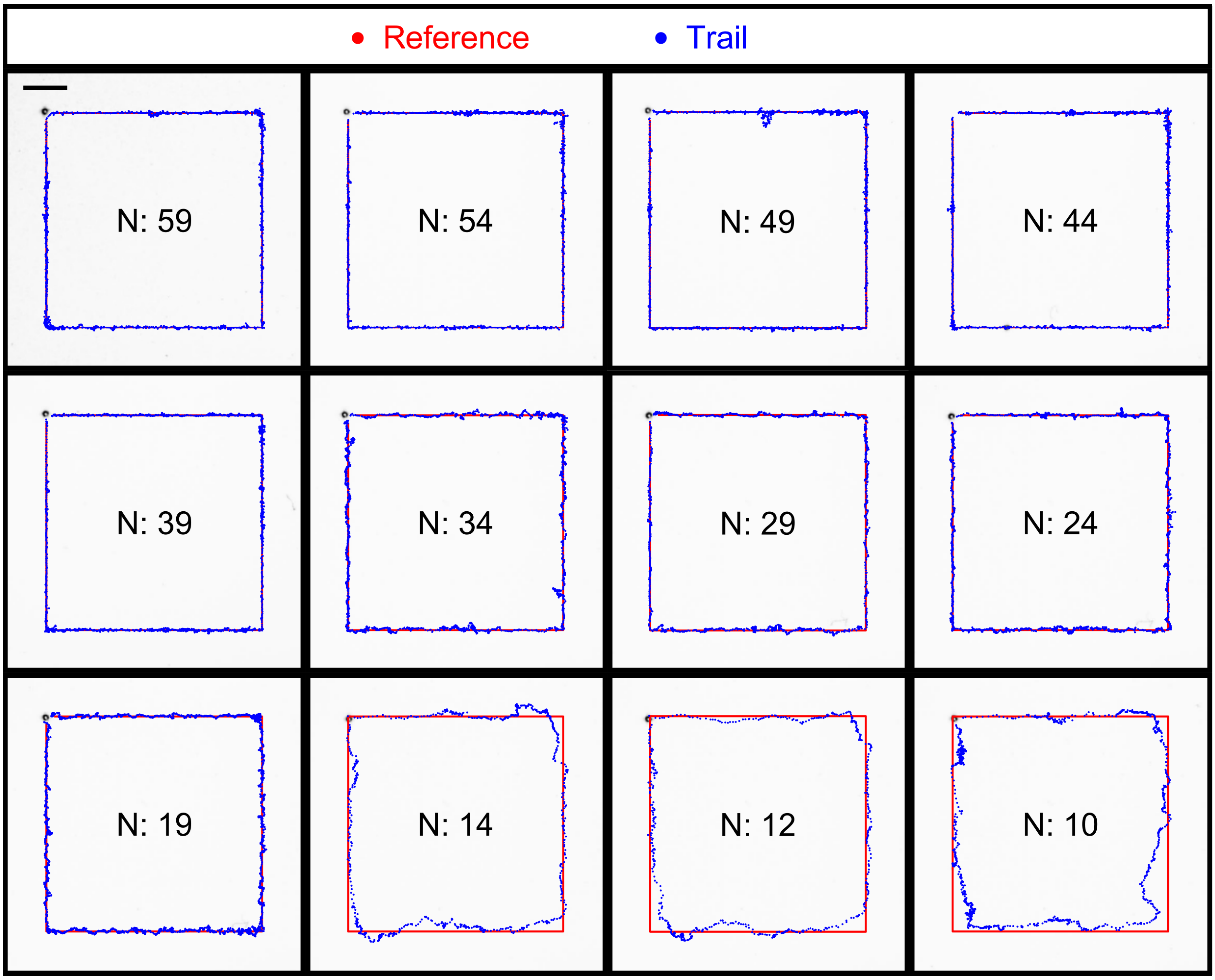

Supplementary Figure 4. Trajectories of line tracking at different number of notes (N). Scale bar: 5 mm.

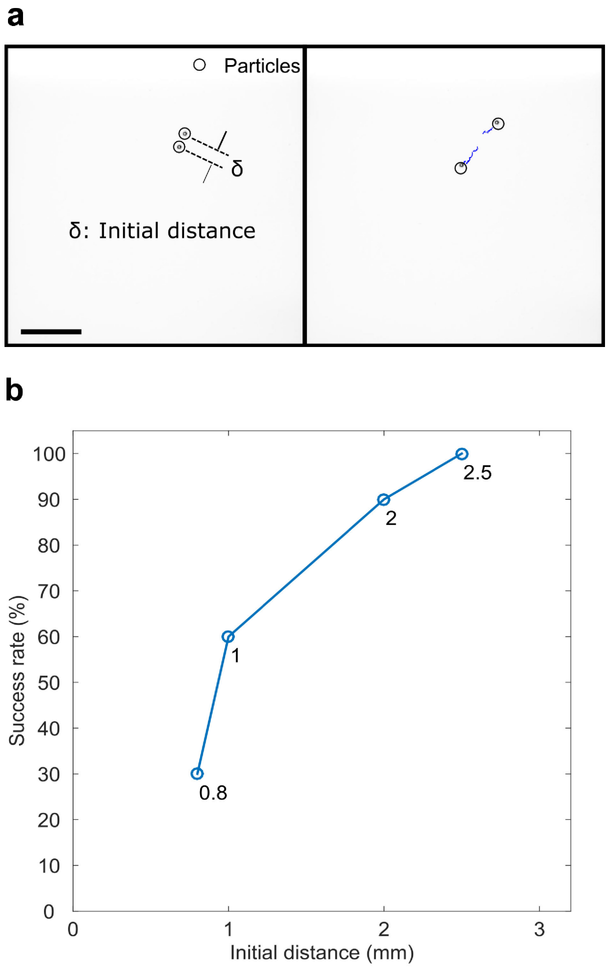

Supplementary Figure 5. Separation of two adjacent particles. (**a**) Illustration of separation experiments. (**b**) The dependency of separation success rate on the initial centre-to-centre distance (δ).

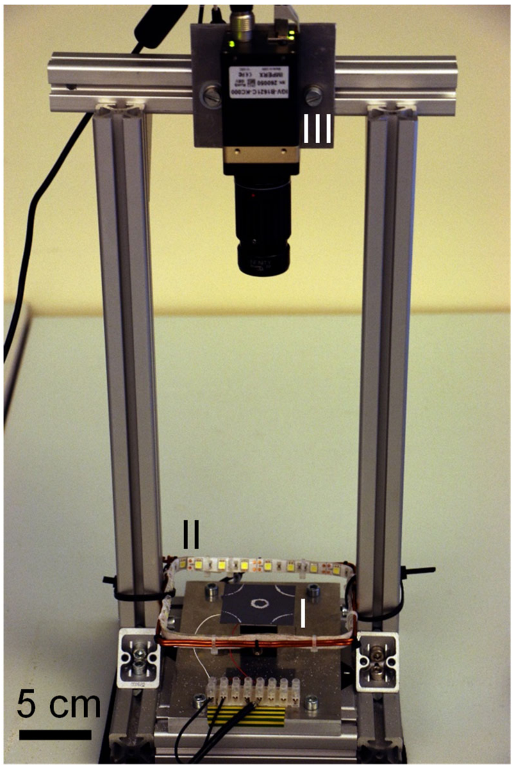

Supplementary Figure 6. Photograph of the experimental setup. I: plate, II: LED-illumination, III: camera.

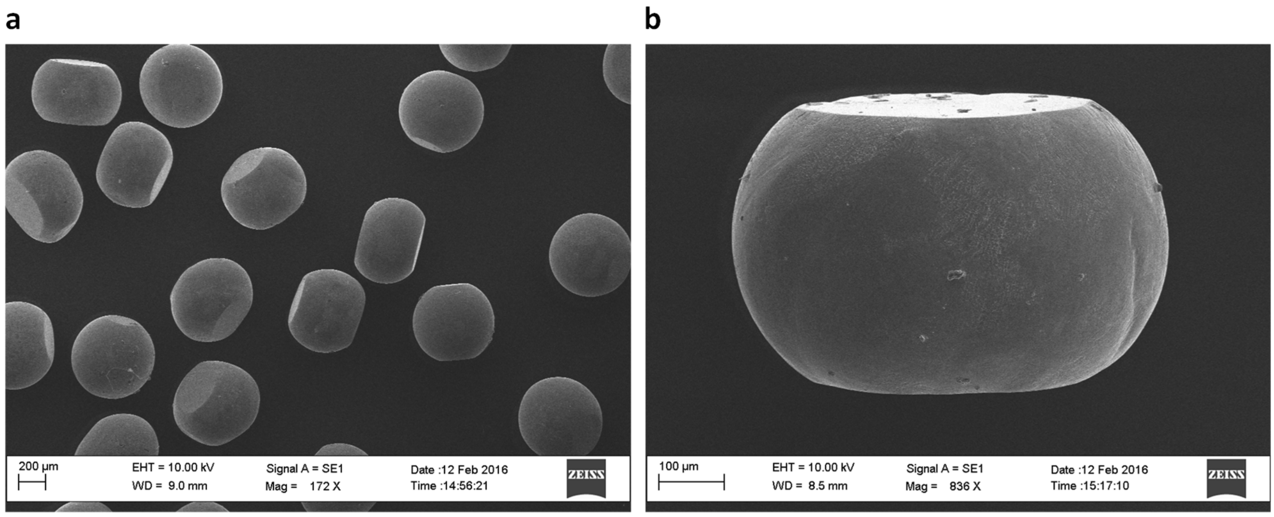

Supplementary Figure 7. SEM micrographs of solder balls. (**a**) shows overview; (**b**) shows close-up of a representative sample.

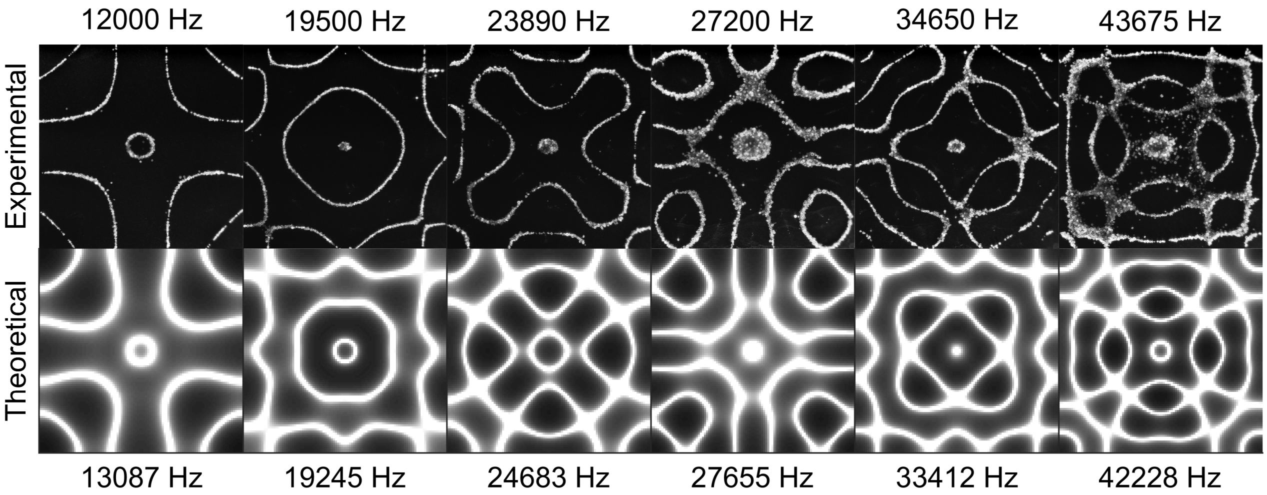

Supplementary Figure 8. Comparison of experimental and theoretical Chladni figures produced by the plate. The theoretical frequencies are the peaks in the entropy function (Supplementary Fig. 4), related to frequency by equation (2) with $C_{\exp}$.

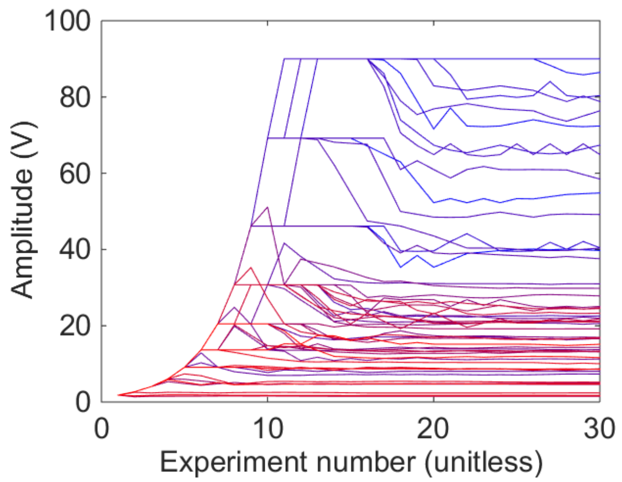

Supplementary Figure 9. Convergence of the nominal amplitude search. The nominal amplitude was searched for 59 notes. The colour of the note corresponds to the frequency (blue = low frequency, red = high frequency).

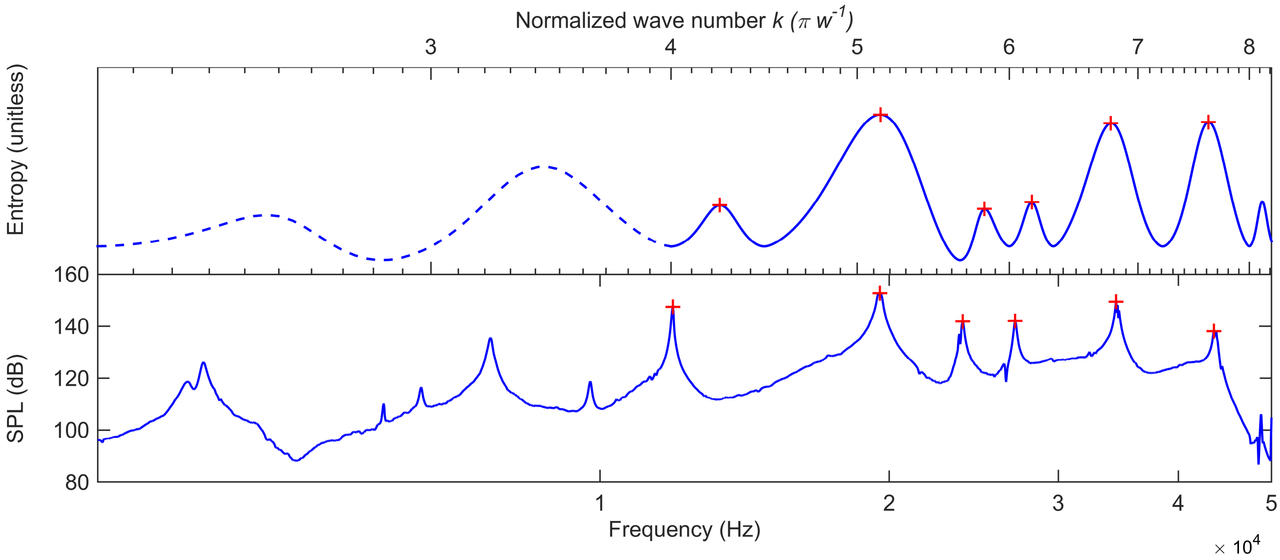

Supplementary Figure 10. Finding resonant peaks theoretically and experimentally. The frequencies were related to the wave number through C_{theor} and eq. (2) and the corresponding peaks were found from both the entropy curve and the acoustic measurement.

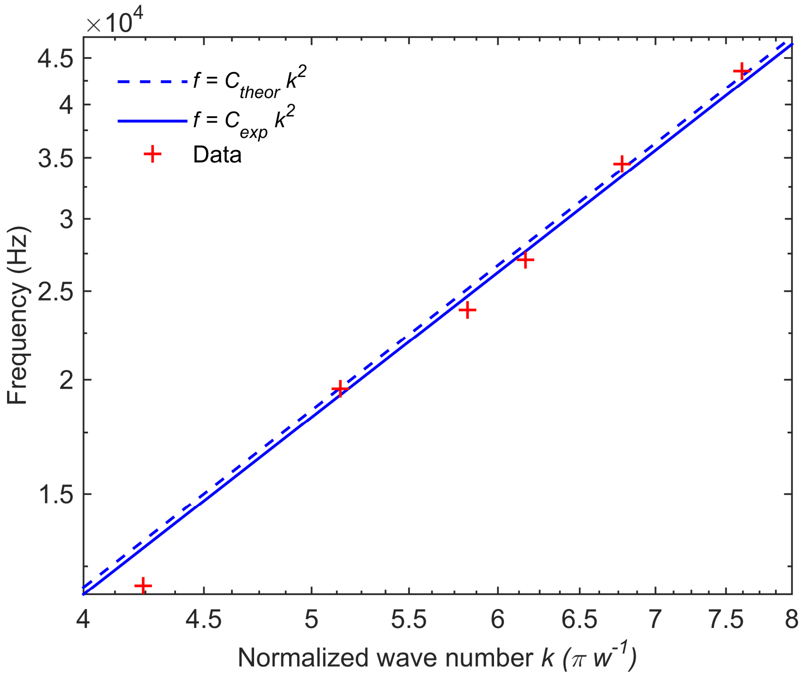

Supplementary Figure 11. Log-log plot comparing the theoretical and experimental dispersion curve for the resonance peaks. The data points are the peaks detected from Supplementary Fig. 7.

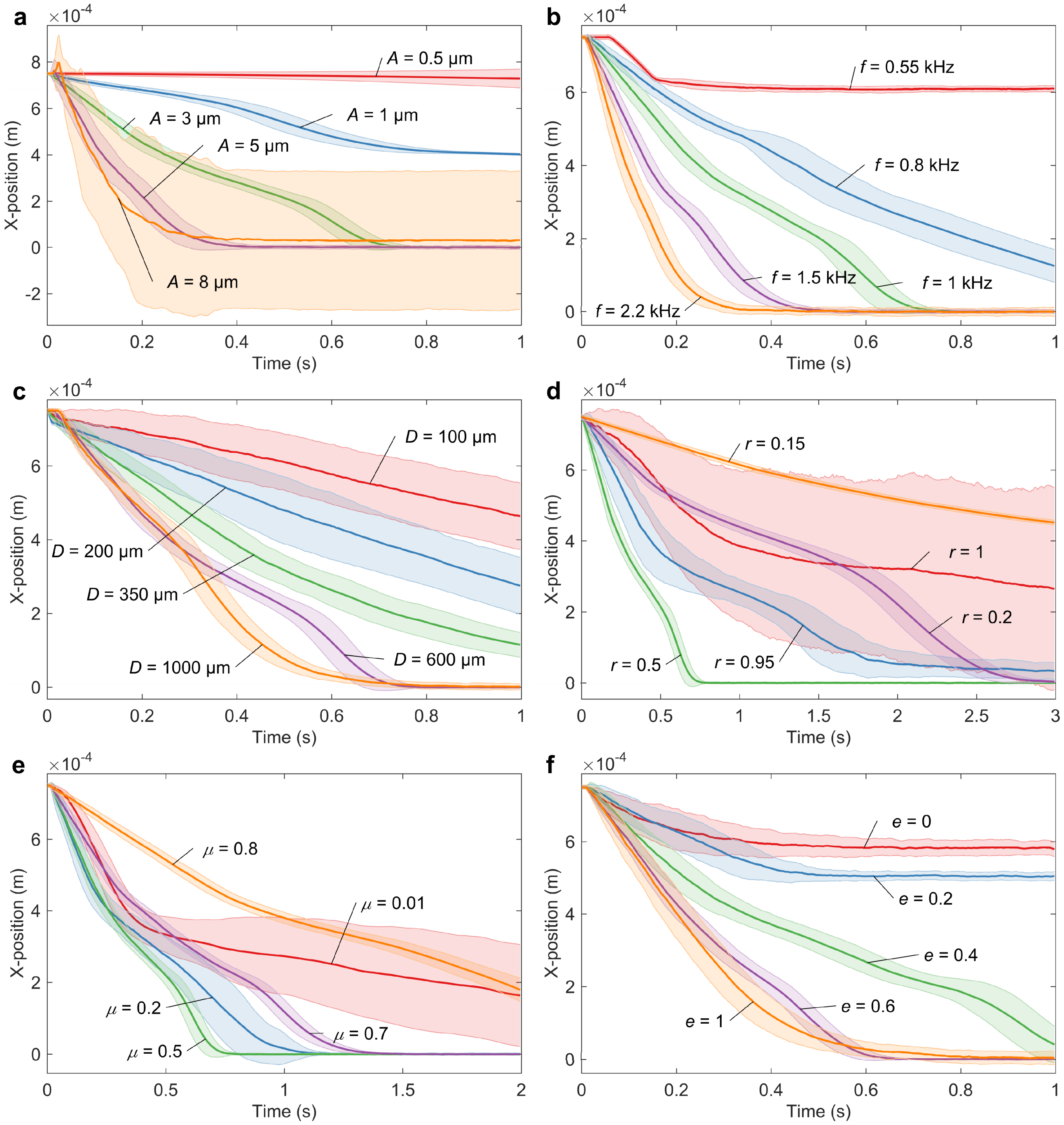

Supplementary Figure 12. Simulation results with different parameters varied. (**a**) vibration amplitude *A*, (**b**) frequency *f*, (**c**) object diameter *D* (aspect ratio kept constant), (**d**) aspect ratio $r = H/D$ (volume kept constant) (**e**) friction coefficient μ (**f**) restitution coefficient *e*.

Supplementary Note 1. Effects of system parameters on object transport using simulations.

Using the developed simulator, we studied the effects of following parameters: vibration amplitude A, friction coefficient μ, restitution coefficient e, aspect ratio $r = H/D$ (while keeping the total object volume constant), and vibration frequency f (while keeping the amplitude and wavelength constant). We performed 100 simulations with each combination of parameters and varied the initial lateral position within a range of ±10 μm, which is close to the resolution of our microscope. From the 100 trajectories, we computed mean trajectory with standard deviations. The simulation results show that:

A. Larger vibration amplitudes, in general, resulted in faster convergence towards the node (Supplementary Fig. 12a). However, larger amplitudes also resulted in larger standard deviations. Eventually the amplitude is large enough that the object can jump over an antinode and converge to another node, resulting in very unpredictable motions for long time horizons (e.g. $A = 8$ μm in Supplementary Fig. 12a).

B. Higher frequency resulted in faster convergence (Supplementary Fig. 12b).

C. Large objects converge faster than small objects (Supplementary Fig. 12c). Thus, to scale the manipulation towards smaller objects, other parameter(s) need to be increased, e.g. restitution, frequency or wave number, if the speed has to be kept constant. However, typically smaller objects are moved shorter distances, so whether speed has to be the same depends on the manipulation task. For large object limit, note that our simulator assumes that the plate is a rigid wall i.e. the mass of the object is insignificant compared to the plate. Very large loads (masses comparable to the mass of the plate) should dampen the vibration, so our simulator likely overestimates the movement for very large objects.

D. Moderate aspect ratio (r = 0.5) resulted in fastest convergence with moderate standard deviations (Supplementary Fig. 12d). Large aspect ratio of $r = 1$ resulted in larger standard deviations, while small aspect ratio of $r = 0.2$ resulted in slower movement.

E. Moderate friction coefficient (μ = 0.5, Supplementary Fig. 12e) resulted in the fastest convergence towards the node. Small friction ($\mu = 0.01$) resulted in uncontrollable sliding, and the standard deviation was larger, i.e. the motion was less predictable. On the other hand, large friction (μ = 0.8) resulted in slower movement, but also smaller standard deviations.

F. Higher restitution resulted in faster convergence (Supplementary Fig. 12f)

These results should be interpreted with caution. We have observed that the system is very sensitive to its parameters and sometimes qualitatively different motions are obtained with only slightly different parameters. This is the case in Supplementary Fig. 12a, where the largest amplitude was sufficient to make the object jump to another node. Thus, using these results to extrapolate far from our values is ill-advised. Another reason to exercise caution is that since the system is nonlinear and chaotic, changing several parameters at the same time might produce qualitatively different results from changing only one at a time. We only varied one parameter at a time due to practical difficulties in testing all the possible parameter combinations exhaustively.
